# Stocking activities for the Arctic charr in Lake Geneva: Genetic effects in space and time

**DOI:** 10.1002/ece3.3073

**Published:** 2017-06-07

**Authors:** Romain Savary, Christophe Dufresnes, Alexis Champigneulle, Arnaud Caudron, Sylvain Dubey, Nicolas Perrin, Luca Fumagalli

**Affiliations:** ^1^ Laboratory for Conservation Biology Department of Ecology and Evolution Biophore University of Lausanne Lausanne Switzerland; ^2^ UMR CARRTEL INRA‐Université de Savoie Mont Blanc Thonon‐les‐Bains Cedex France; ^3^ Science‐Management Interface for Biodiversity Conservation Thonon‐les‐Bains France; ^4^ Hintermann & Weber SA Montreux Switzerland

**Keywords:** conservation‐based stocking programs, fisheries management, genetic bottleneck, genetic integrity, historical DNA, microsatellites, Salmonids, *Salvelinus*

## Abstract

Artificial stocking practices are widely used by resource managers worldwide, in order to sustain fish populations exploited by both recreational and commercial activities, but their benefits are controversial. Former practices involved exotic strains, although current programs rather consider artificial breeding of local fishes (supportive breeding). Understanding the complex genetic effects of these management strategies is an important challenge with economic and conservation implications, especially in the context of population declines. In this study, we focus on the declining Arctic charr (*Salvelinus alpinus*) population from Lake Geneva (Switzerland and France), which has initially been restocked with allochtonous fishes in the early eighties, followed by supportive breeding. In this context, we conducted a genetic survey to document the evolution of the genetic diversity and structure throughout the last 50 years, before and after the initiation of hatchery supplementation, using contemporary and historical samples. We show that the introduction of exotic fishes was associated with a genetic bottleneck in the 1980–1990s, a break of Hardy–Weinberg Equilibrium (HWE), a reduction in genetic diversity, an increase in genetic structure among spawning sites, and a change in their genetic composition. Together with better environmental conditions, three decades of subsequent supportive breeding using local fishes allowed to re‐establish HWE and the initial levels of genetic variation. However, current spawning sites have not fully recovered their original genetic composition and were extensively homogenized across the lake. Our study demonstrates the drastic genetic consequences of different restocking tactics in a comprehensive spatiotemporal framework and suggests that genetic alteration by nonlocal stocking may be partly reversible through supportive breeding. We recommend that conservation‐based programs consider local diversity and implement adequate protocols to limit the genetic homogenization of this Arctic charr population.

## 1 INTRODUCTION

Wildlife translocation programs involving captive breeding are common in the conservation context (e.g., reintroduction plans), as well as in a wide array of industries, such as forestry, agriculture, ranching, and especially aquaculture. Yet, the artificial propagation of exogenous wildlife can pose a severe threat on local biodiversity, leading to population declines and biological invasions (Laikre, Schwartz, Waples, Ryman, & Ge, 2010). In addition, several genetic issues might affect the success of captive breeding programs (reviewed in Frankham, Ballou, & Briscoe, 2010). Many fish species are massively exploited as food resources, and artificial stocking has been widely applied by the fishing industry to improve productivity. Artificial stocking originally involved large‐scale releases of non‐native individuals during the XIXth and most of the XXth century, and more recently conservation‐based supportive breeding programs (i.e., captive breeding of a fraction of the local population followed by its release into the wild). An array of detrimental effects is known to result from these controversial practices. Large‐scale releases of genetically impoverished captive fishes can cause changes in population composition and structure in the wild, as well as losses of genetic diversity due to a reduction in effective population size (N_e_) (e.g., Allendorf, Berry, & Ryman, 2014; Allendorf, Luikart, & Aitken, 2012; Hansen, Nielsen, Ruzzante, Bouza, & Mensberg, 2000; Ryman & Laikre, 1991; Wang & Ryman, 2001; Waples & Do, 1994). Moreover, introgressive hybridization with hatchery‐reared individuals may decrease the fitness of local populations due to the replacement of locally adapted alleles with exotic, nonadaptive ones, or because artificially reared individuals from local origin have become maladapted to wild conditions as a result of domestication selection (Allendorf, England, Luikart, Ritchie, & Ryman, 2008; Araki, Berejikian, Ford, & Blouin, 2008; Araki, Cooper, & Blouin, 2007, 2009; Araki & Schmid, 2010; Baskett & Waples, 2012; Christie, Ford, & Blouin, 2014; Frankham et al., 2010; Fraser, Weir, Bernatchez, Hansen, & Taylor, 2011; Garcia de Leaniz et al., 2007; Hansen et al., 2000; Laikre & Rymand, 1996; Laikre et al., 2010). Numerous studies documented extensive genetic homogenization of native populations due to large‐scale interbreeding with hatchery strains (e.g., Araki & Schmid, 2010; Ayllon, Martinez, & Garciavazquez, 2006; Bartron & Scribner, 2004; Eldridge, Myers, & Naish, 2009; Lamaze, Sauvage, Marie, Garant, & Bernatchez, 2012; Largiadèr & Scholl, 1995; Marie, Bernatchez, & Garant, 2010; Perrier, Guyomard, Bagliniere, Nikolic, & Evanno, 2013; Vasemägi, Gross, Paaver, Koljonen, & Nilsson, 2005). Note that under certain circumstances genetic diversity can locally increase when new alleles are introduced by allochtonous individuals, eventually leading to outbreeding depression as adapted gene–gene or gene–environmental interactions are disrupted (Allendorf, Leary, Spruell, & Wenburg, 2001; Bougas, Audet, & Bernatchez, 2010; Ciborowski et al., 2007; Edmands, 2007; Le Cam, Perrier, Besnard, Bernatchez, & Evanno, 2015; Marie et al., 2010). Careless traditional stocking practices can thus profoundly hinder the genetic integrity of wild populations, which may in turn impact their long‐term survival.

In contrast, supportive breeding strategies using annually collected native broodstock (gametes) and the implementation of protocols accounting for broodstock numbers, relatedness, sex‐ratio, and life‐history traits can minimize such adverse effects (e.g., Eldridge & Killebrew, 2008; Fraser, 2008; Hess et al., 2012; Milot, Perrier, Papillon, Dodson, & Bernatchez, 2013; Wang & Ryman, 2001; Wedekind, 2002). Two types of supportive breeding are usually applied, involving either offspring from wild caught individuals or progeny (F2) of these offspring. These protocols are increasingly used in conservation‐based stocking strategies to maintain high‐density, self‐sustaining wild populations (Adkison, 1995; Caughley & Gunn, 1996; Heggenes, Beere, Tamkee, & Taylor, 2006). To date, few studies have comprehensively monitored the long‐term genetic consequences of stocking, especially when the two main strategies (traditional restocking with non‐native fishes vs supportive breeding) were applied in turns. Historical samples can provide valuable information for such questions. In many salmonid populations, scales have been archived for decades, initially for age determination and population monitoring purposes, but they now also provide reliable sources of DNA for retrospective genetic analysis (e.g., Bonanomi et al., 2015; Nielsen & Hansen, 2008; Nielsen, Hansen, & Loeschcke, 1999).

Fishes of the family Salmonidae are well‐known for their potential to evolve small‐scale genetic structures, which are often linked to geographical landscapes (e.g., Bernatchez, Dempson, & Martin, 1998; Corrigan, Lucas, Winfield, & Hoelzel, 2011; Stelkens, Jaffuel, Escher, & Wedekind, 2012; Taylor, 1991). Accordingly, they often exhibit a mosaic of morphological traits, breeding tactics, and behavior even at the intra‐specific level (Balon, 1980; Moore & Bronte, 2001; Reist, Gyselman, Babaluk, Johnson, & Wissink, 1995; Taylor, 1991) and hence are particularly well adapted to narrow ecological niches. The maintenance of their genetic variability is therefore of main importance for their adaptive potential and conservation. As such, this group is highly sensitive to the effects of stocking practices (Araki & Schmid, 2010).

The Arctic charr (*Salvelinus alpinus*) is a circumpolar stenothermal cold water salmonid. This species demonstrates a high potential for local adaptation, which can lead to genetically and ecologically divergent sympatric morphs (Corrigan et al., 2011; Gomez‐Uchida, Dunphy, O'Connell, & Ruzzante, 2008; Skoglund, Siwertsson, Amundsen, & Knudsen, 2015; Westgaard, Klemetsen, & Knudsen, 2004). For instance, up to four sympatric morphotypes are found in the single lake Thingvallavatn in Iceland (Sandlund et al., 1992). The Arctic charr is native to Lake Geneva (Switzerland/France), which corresponds to the southern boundary of its natural European distribution. It spawns in deep areas (40–120 m) with low sedimentation, is highly sensitive to changes in oxygen levels and temperature, and is therefore potentially affected by eutrophication and global warming (Mari, Garaud, Evanno, & Lasne, 2016). Quality and quantity of available spawning sites are limiting factors in this lake (Rubin, 1990).

The Arctic charr population of Lake Geneva provides a unique opportunity to document the genetic impact of both traditional stocking and supportive breeding strategies in a spatiotemporal framework. At the end of the 1970s, a massive restocking program was initiated, first based on allochtonous eggs imported from Denmark (~600,000 domestic juveniles released from 1979 to 1982; Rubin, 2005; Champigneulle, comm. pers.) and then followed by a conservation‐orientated strategy restricted to juveniles obtained from artificially fertilized eggs of wild spawners from the lake. The latter involved an increasing amount of yearly releases, from 150,000 in the 1980s to 1,600,000 at present (Champigneulle & Gerdeaux, 1995; Rubin, 2005; detailed in Caudron, Lasne, Gillet, Guillard, & Champigneulle, 2014). The intense restocking program rapidly promoted catches up until the late 1990s, but these abruptly decreased from 1999 onwards, despite 30 years of habitat quality recovery (reoligotrophication; Caudron et al., 2014). The current inefficiency of the ongoing supportive breeding program was further supported by capture–mark–recapture studies (Caudron et al., 2014), raising important concerns about the self‐sustainability of the Arctic charr population (less than half of caught adults are stocked fishes). Several hypotheses have been proposed to explain this decline, namely inter‐ and intraspecific competition, predation by pike, diseases, maladapted stock management, environmental changes, and/or genetic effects of the long‐term stocking (Caudron et al., 2014).

In this study, we take advantage of historical and contemporary samples to document the long‐term consequences of 30 years of stocking activities on the genetic composition, structure, and diversity of the Arctic charr population of Lake Geneva, in a context of recent declines. We genotyped fast‐evolving microsatellite markers in both contemporary and historical (archived scales) samples covering more than 50 years of fishing history from different parts of the lake. Our results have strong relevance for the design of future management strategies for this species.

## 2 METHODS

### 2.1 Sampling and DNA extraction

**Figure 1:**
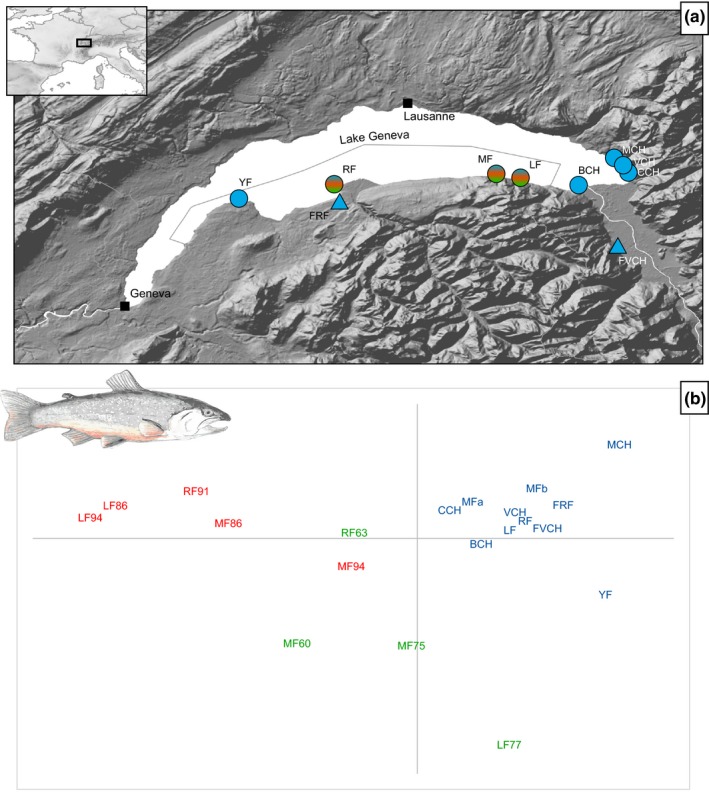
(a) Study area and sampling sites and (b) Principal Component Analysis (PCA) on population allele frequency. Colors discriminate the three main time periods: 1960–1970s, prior to the stocking programs (green); 1980–1990s, in early years of the stocking programs (red), and present time (blue). On the map, circles, and triangles indicate natural spawning sites and fish farms, respectively. On the PCA, axes 1 and 2 account for 26.7% and 14.3%, respectively, but only the first axis is significant (*p *<* *.05). Drawing: Romain Savary

We included a total of 378 individuals from the eight known natural spawning sites of Lake Geneva (Brunner, Douglas, & Bernatchez, 1998; Rubin, 2005), as well as the two fish farms involved in current supportive breeding activities (Table 1, Figure 1). Contemporary samples (*n* = 218, coded in blue color throughout the figures) were collected in 2007 (fish farms) and during the winter 2009–2010 (natural spawning sites) when the fishes breed. Tissue samples were obtained from adipose fins (wild adults, captured in December 2009 as part of the supportive breeding program) or tail tips (juveniles in fish farms), stored in 80% ethanol. One site (MF) was sampled first in December 2009 (MFa) and a second time later in February 2010 (MFb), to control that fishes are not genetically structured locally due to disruptive breeding periods among individuals. For three sites (RF, MF, LF), we gathered series of historical samples (*n* = 160 dried scales collected by local fishermen and researchers) from two main time periods: before (1960–1977, *n* = 61 from four series, coded in green) and few years after the beginning of the stocking program in 1979 (1986–1994, *n* = 99 from five series, coded in red).

**Table 1:** Sampling details of Arctic charr contemporary and historical samples

Site	Latitude	Longitude	Code	Date	*n*
Contemporary samples					
Chillon	46.4140	6.9249	CCH	December 2009	20
La Veraye	46.4250	6.9160	VCH	December 2009	20
Bay of Montreux	46.4363	6.9015	MCH	December 2009	20
Bouveret	46.3942	6.8485	BCH	December 2009	20
Yvoire	46.3737	6.3265	YF	December 2009	20
Ripaille	46.3957	6.4718	RF	December 2009	20
Locum	46.4049	6.7588	LF	December 2009	19
Meillerie	46.4113	6.7216	MFa	December 2009	20
			MFb	February 2010	20
Fish farm of Vouvry	46.2983	6.9080	FVCH	2007	19
Fish farm of Rives	46.3663	6.4801	FRF	2007	20
Historical samples					
Ripaille	46.3957	6.4718	RF63	1963	12
			RF91	1991	20
Locum	46.4049	6.7588	LF77	1977	12
			LF86	1986	20
			LF94	1994	20
Meillerie	46.4113	6.7216	MF60	1960	17
			MF75	1975	20
			MF86	1986	20
			MF94	1994	19

DNA was extracted with the Qiagen BioSprint 96 extraction robot (contemporary samples) or the Qiagen DNAeasy Blood & Tissue Extraction kit (historical samples). Historical samples (~10 scales per individual) were processed in a separate laboratory dedicated only to the extraction of low copy‐number DNA samples and preparation of pre‐PCR reagents. Negative controls were employed during all extraction and amplification experiments.

### 2.2 Microsatellite genotyping

We analyzed eight microsatellite markers polymorphic in the Arctic charr (Bernatchez et al., 1998; Brunner et al., 1998) (listed in Table S1, with their PCR conditions). For contemporary samples, PCR cycles were as follows: initial denaturation at 94°C for 5′; locus‐specific number of PCR cycles (Table S1) consisting of denaturation at 94°C for 30″, annealing at a locus‐specific temperature for 30″ (Table S1), and extension at 72°C for 30″; final extension at 72°C for 7″. We used a similar protocol for historical samples, but with 50 PCR cycles in all instances. Amplicons of each marker were pooled in three groups for multiplex genotyping (Table S1) and run on an ABI3100 sequencer (Applied Biosystems).

In order to account for allelic dropouts due to low DNA‐content, each historical sample was independently genotyped four times to obtain a consensus genotype, which appeared to be reliable based on preliminary tests. The only exception was one sample series consisting of chemically cleaned scales (RF63), which was genotyped eight times.

### 2.3 Population genetic analyses

Genotypes were preliminary checked for allele dropout using Micro‐Checker (Van Oosterhout, Hutchinson, Wills, & Shipley, 2004). We estimated the genetic diversity of each site/sample series by computing the observed heterozygosity (*H*_o_), allelic richness (*A*_r_), and inbreeding coefficient (*F*_is_) in Fstat (Goudet, 1995). The genetic structure was assessed in four ways. First, we performed a PCA based on population allele frequencies with PCAgen (Goudet, 2002), testing for the significance of axes by 10,000 permutations. Second, we calculated pairwise genetic differentiation (*F*_st_) in Fstat, and used TreeFit (Kalinowski, 2009) to build a neighbor‐joining tree of genetic distances based on these *F*_st_. Third, we performed an analysis of molecular variance (AMOVA) in Arlequin 3.5 (Excoffier, Laval, & Schneider, 2005) to understand how the genetic variation was shaped temporally (among the three time periods) and spatially (among sites). Finally, we attempted to assign individual genotypes into groups using the Bayesian clustering algorithm of STRUCTURE (Pritchard, Stephens, & Donnelly, 2000), testing from *K* = 1 to 10 with 100,000 iterations after a burnin of 10,000. Graphic (boxplots) and statistical comparisons (nonparametric analyses of variance, ANOVA, with 10,000 bootstrap replicates) were performed in R (R Development Core Team, 2011).

## 3 RESULTS

### 3.1 Microsatellite variability

Our microsatellites were highly polymorphic in the studied population, with an average heterozygosity (*H*_o_) of 0.65 among spawning sites (0.16–0.95) and a total number of alleles per marker (k) of 15 (5–29). Signs of null alleles or allele dropouts were detected in only nine of 160 instances, involving both contemporary (five instances) and historical samples (four instances), and mainly affecting two loci: Sfo23 (three instances, sites FVCH, VCH and MF94) and MTS‐85 (four instances: MFb, MF75, MF94, LF86). Therefore, this might rather reflect true null alleles segregating at low frequencies, or disequilibrium resulting from drift, rather than dropout due to degraded historical DNA. Details on each locus are provided in Table S2.

### 3.2 Patterns of genetic structure

**Figure 2:**
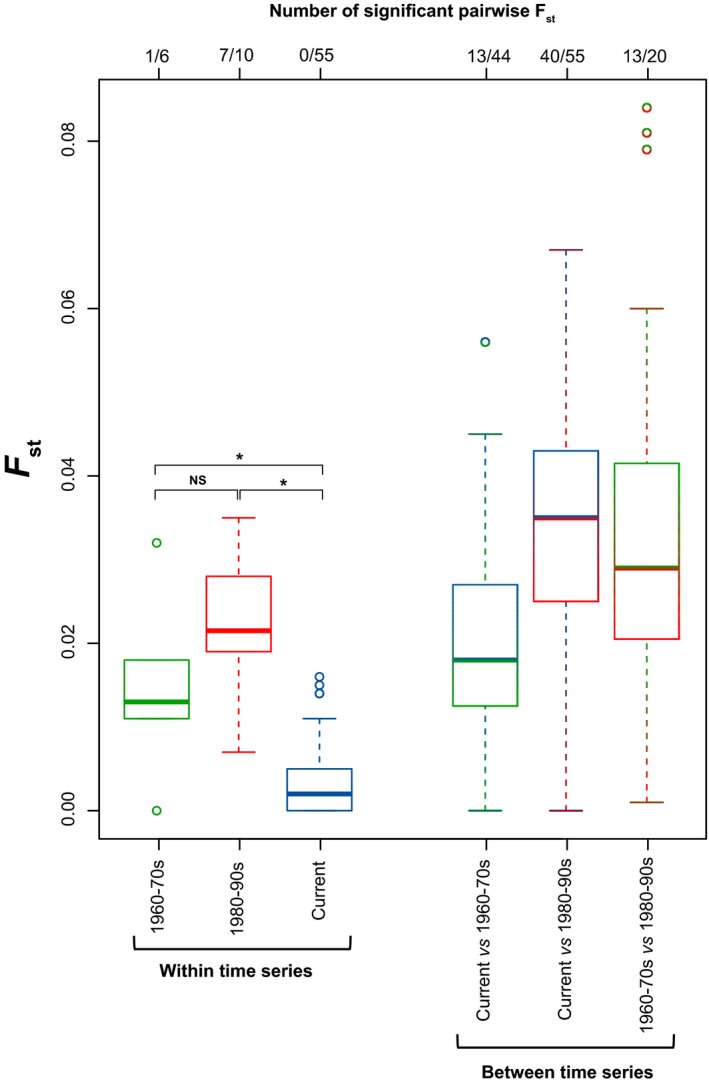
Genetic structure over the lake for each time period (left) and change in genetic composition between time periods (right), computed from pairwise *F*_st_. Number of comparisons, and how many significantly depart from 0, are indicated on top. Differences between the main time periods were statistically tested by nonparametric ANOVAs with 10,000 permutations. NS: nonsignificant; *: *p *<* *.05. In boxplots, bands represent medians; boxes represent interquartile range of data (IQR); whiskers represent minimum and maximum values, or to 1.5 times the IQR, whichever is the smaller; circles represent outliers

The genetic structure of Lake Geneva's population varied from weak to virtually null throughout the last 50 years (Figure 2, left). Most of the genetic variance was found at the individual level (Table 2). Pairwise *F*_st_ were slightly higher after (1980–1990s; *F*_st_ = 0.022 ± 0.007, significantly departing from 0 in seven out of ten comparisons) than before the initiation of the traditional restocking program (1960–70s; *F*_st_ = 0.015 ± 0.010, significantly departing from 0 in one of six comparisons). Contemporary sites, including the two hatcheries, were not genetically structured (*F*_st_ = 0.004 ± 0.005, never significantly departing from 0). These patterns are also illustrated by the PCA on allele frequency (narrow clustering of contemporary samples compared to historical ones; Figure 1b) and the tree of genetic distance (shorter, closely‐related branches for contemporary samples; Fig. S1).

**Table 2:** Analysis of Molecular Variance (AMOVA) in the Arctic charr population of Lake Geneva

Source of variation	Sum of squares	Variance components	F	% variation
Among periods	28.8	0.051	0.019	1.9
Among sites within period	59.8	0.023	0.009	0.9
Within sites	1935.7	2.630	0.027	97.2

F statistics (fixation indices) correspond to *F*_st_ (among sites among periods), *F*_sc_ (among sites within periods), *F*_ct_ (among periods).

We detected some genetic differentiation between present and original fishes (i.e., prior stocking) (*F*_st_ = 0.020 ± 0.012, departing from 0 in 13 of 44 comparisons; Figure 2 right). The displacement was stronger between fishes caught at the beginning of the stocking programs and those caught recently (*F*_st_ = 0.033 ± 0.017, departing from 0 in 40 of 55 comparisons), as well as compared to original ones (*F*_st_ = 0.035 ± 0.024, departing from 0 in 13 of 20 comparisons). Accordingly, the first axis of the populational PCA (accounting for 26.7% of the total variance), and the tree of genetic distances mainly distinguished samples according to their time series rather than their geographical origin (Figure 1b, Fig. S1). Clustering with STRUCTURE did not yield any intelligible solution (not shown), suggesting no significant structure and a nontestable solution of *K* = 1.

### 3.3 Patterns of genetic diversity

**Figure 3:**
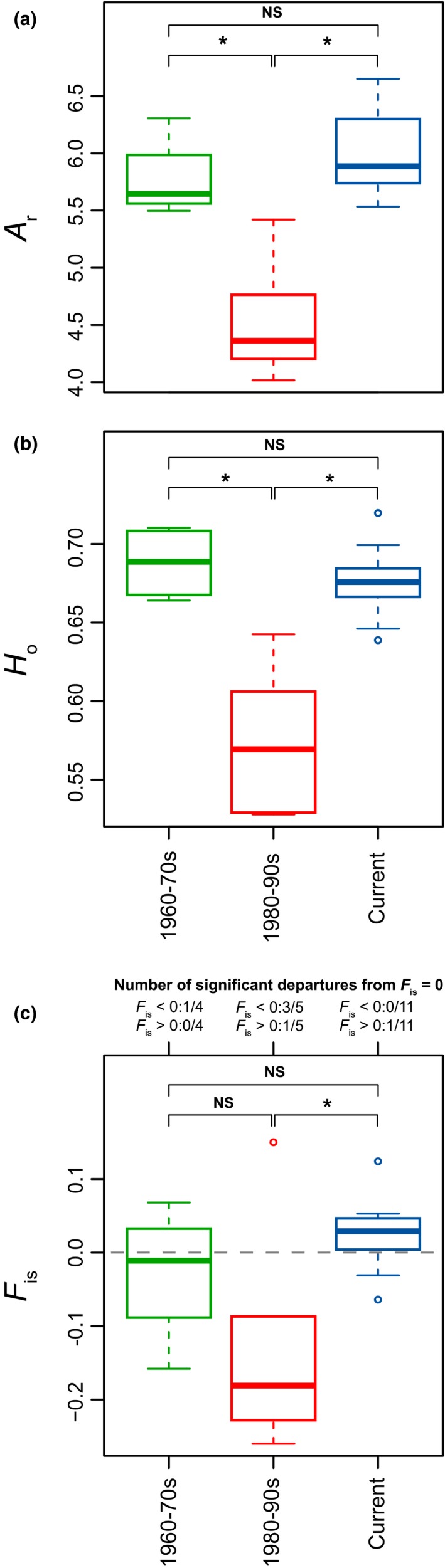
Variation in (a) Allelic richness (*A*_r_), (b) observed heterozygosity (*H*_o_), and (c) inbreeding coefficient (*F*_is_) in the three main time period. Significance of statistical comparisons (nonparametric ANOVA with 10,000 permutations) are given (NS: nonsignificant; *: *p *<* *.05). For (c), the number of *F*_is_ coefficient significantly departing from HWE (*F*_is_ = 0) are given for each period. In boxplots, bands represent medians; boxes represent interquartile range of data (IQR); whiskers represent minimum and maximum values, or to 1.5 times the IQR, whichever is the smaller; circles represent outliers

Allelic richness (*A*_r_, scaled to nine individuals) and observed heterozygosity (*H*_o_) were significantly lower in the early years of stocking (*A*_r_ = 4.6 ± 0.56, *H*_o_ = 0.57 ± 0.05) than prior to it (*A*_r_ = 5.8 ± 0.36, *H*_o_ = 0.69 ± 0.02), and nowadays (*A*_r_ = 6.0 ± 0.37, *H*_o_ = 0.68 ± 0.02) (Figure 3a and b). Interestingly, most current and original sites were at Hardy–Weinberg Equilibrium (HWE; *F*_is_ close to, and not significantly departing from 0), but sites from the 1980 to 1990s show significant excesses of heterozygotes compared to HWE in three of five instances (Figure 3c). Fishes collected from the two hatcheries featured similar levels of diversity that fishes collected in the lake (for FVCH, *A*_r_ = 6.6, *H*_o_ = 0.70; for FRF, *A*_r_ = 5.9, *H*_o_ = 0.67), but one hatchery (FVCH) shows a clear deficit of heterozygotes (*F*_is_ = 0.124, the only contemporary site with a significant *F*_is_).

### 3.4 Fine‐scale genetic changes over time

**Figure 4:**
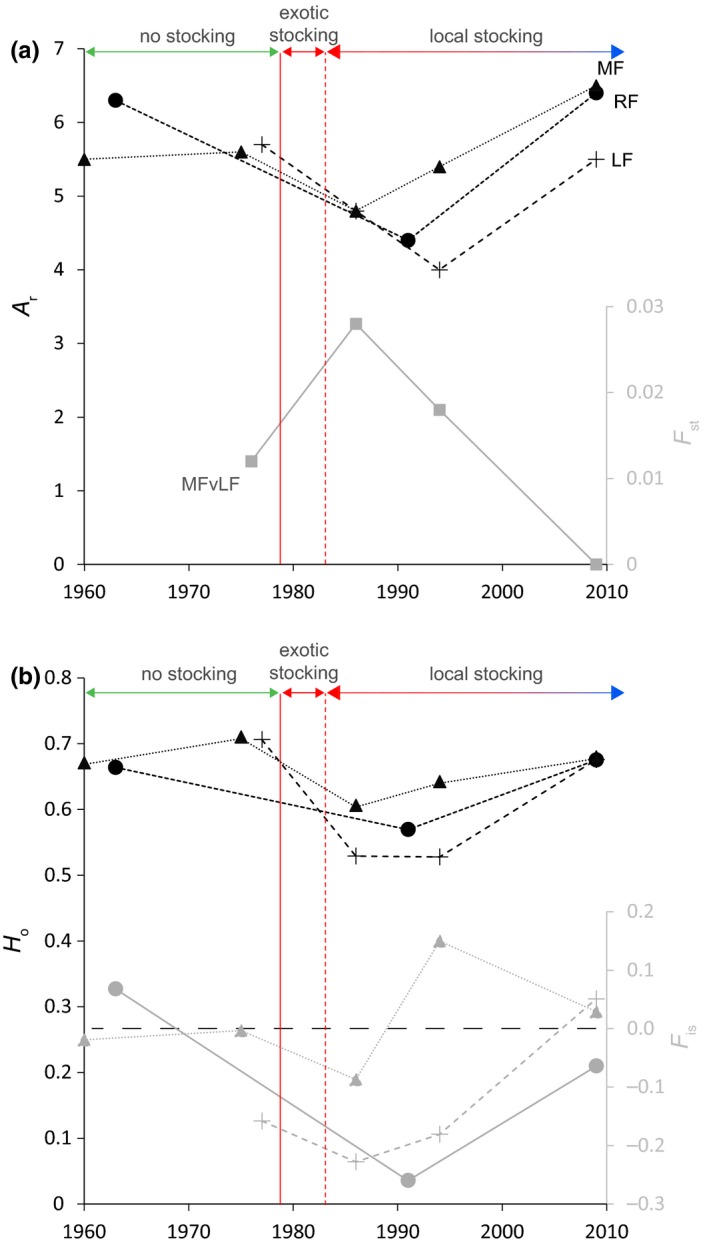
Evolution of (a) allelic richness (*A*_r_), population differentiation (*F*_st_), (b) observed heterozygosity (*H*_o_), and inbreeding coefficient (*F*_is_) over time, for the three sampling sites where time series are available. For *F*_st_, only the differentiation between MF and LF is shown, given that historic samples from the same (1963, 1986, 1994) or close years (1975 and 1977) were only available for these two sites. The colored arrows indicate areas before the stocking programs (no stocking), in early stages of the traditional restocking involving exotic fishes from Denmark (1979–1982) (exotic stocking), and the following years till nowadays, involving supportive breeding with local fishes (local stocking). Crosses: LF, circles: RF, triangles: MF; squares: genetic differentiation (*F*_st_) between MF and LF

Closer inspection of the three spawning sites for which genetic data was available at several time points (MF, RF, and LF) confirmed the general patterns observed over the main time periods (Figure 4). For all three sites, *A*_r_ and *H*_o_ dropped in the years following traditional stocking with nonlocal fishes, which was paralleled by an increase of genetic structure (*F*_st_) and outbreeding (negative *F*_is_). During the period of supportive breeding (1983‐present), initial diversity levels and HWE were restored and genetic structure was suppressed.

## 4 DISCUSSION

Our study provides a comprehensive temporal and spatial framework to understand the evolution of genetic diversity and structure of the Arctic charr in Lake Geneva, in the context of two different stocking practices applied in turns: the traditional stocking using artificially reared exotic fishes (from Denmark) from 1979 to 1982; followed by the continuous supportive breeding of wild spawners from 1983 until now. However, this intense restocking program did not allow this population to reach self‐sustainability, the species being declining for two decades despite habitat recovery. Our results provide new insights about the controversy of these practices. Importantly, these findings are based on wild spawners, effectively contributing to the next generation.

### 4.1 Genetic effects of the traditional restocking program (1979–1982)

Our analyses demonstrate clear‐cut genetic effects of the stocking programs. The massive releases of Danish fishes (1979–1982) caused significant changes in the genetic structure, nature, and diversity of the lake's population. First, the diversity within sites decreased right after the initiation of the restocking, potentially due to strong founder effects or initially low diversity of the introduced fishes. The common practice is to raise and release the offspring of few adults (artificially obtained by in‐farm fecundation), yielding a disproportionate amount of siblings among released stocks and thus low effective size and genetic diversity. The present pattern supports that very few breeders contributed to the imported eggs or that their diversity was low to begin with. In addition, it is not excluded that demographic processes (i.e., historical decline, as reported during the 1970s in the lake) may have contributed to this genetic bottleneck. Second, despite low intrinsic diversity, these early restocked sites (1980–1990s) are also the only ones with an excess of heterozygotes (negative *F*_is_). This pattern likely reflects a situation where many fishes are the product of admixture between exotic and local fishes (as, e.g., Gharrett, Smoker, Reisenbichler, & Taylor, 1999; Le Cam et al., 2015; Valiquette, Perrier, Thibault, & Bernatchez, 2014), which may have induced outbreeding depression (breakdown of co‐adapted interactions; Allendorf et al., 2001; Edmands, 2007; Bougas et al., 2010). Given that Arctic charrs first reproduce 4–5 years after birth (Jonsson & Hindar, 1982), we assume the Danish gene pool to be detectable among catches from 1983 onwards, a period well‐covered by our historical samples (1986, 1991, 1994). Fishes from this period also seem substantially different than original wild stocks (disruptive clustering on the PCA, Figure 1; significant F_st_ estimates, Figure 2 right). Unfortunately, the Danish source populations no longer exist and no scale sample of the stock fish is available, which would have enabled to estimate their relatedness with Swiss fishes and get better insights into patterns of hybridization. Third, another detectable effect is the increase in overall population structure across the lake. This probably reflects that releases were conducted in few areas and that not all sites were reached by the released gene pool at this period in the same way.

These rapid genetic responses are in line with the majority of studies focusing on stocking with nonautochthonous fishes. These accordingly reported reduction in genetic diversity and significant genetic displacement between original and present stocks (reviewed by Araki & Schmid, 2010). Such practices led to admixture through introgressive hybridization and sometimes up to the complete replacement of the original diversity (Miller, Mero, & Younk, 2012; Perrier, Guyomard, Bagliniere, & Evanno, 2011; Perrier et al., 2013), an effect intimately linked to stocking intensity (Valiquette et al., 2014). In some cases, the genetic differentiation is paralleled by disruptive behaviors, which can thus potentially hinder fitness parameters of restocked populations, especially during reproduction (e.g., Blanchet, Paez, Bernatchez, & Dodson, 2008; Flemming, Lamberg, & Jonsson, 1997; Hansen et al., 2000; Ryman & Stahl, 1980; Verspoor, 1988). Decreases in genetic diversity following restocking are often associated with lower survival and reproductive performance (Araki & Schmid, 2010), although there are some exceptions (Christie et al., 2014). Moreover, salmonid fishes are known to naturally retain strong population structure at the local scale over time, indicative of population stability and potential for local adaptation (see 1). These important features may obviously be compromised by massive restocking from non‐native fishes. In the case of our population, however, the genetic structure across Lake Geneva was weak overall, even prior to the restocking. This original near‐panmixia thus seem in contradiction with the well‐known homing behavior of salmonids (Westgaard et al., 2004) and could be explained by the lack of geographical barriers and the proximity of spawning sites in the lake. It is also not excluded that a stronger structure could have existed previously, although salmonids are known for their high temporal stability (e.g., Gow, Tamkee, Heggenes, Wilson, & Taylor, 2011; Hansen, 2002; Tessier & Bernatchez, 1999; Van Doornik, Waples, Baird, Moran, & Berntson, 2011).

### 4.2 Genetic effects of the conservation‐based program (1983‐)

The detrimental genetic consequences of restocking by allochtonous fishes, now prohibited in most countries (since 1991 in Switzerland, by federal law), have been clearly demonstrated (see 1), and our study brings further support. In contrast, conservation‐based strategies, involving supplementation by local fishes are less controversial, as they do not imply genetic pollution by exotic, potentially maladapted, gene pools. Here, the three decades of supportive breeding, from 1983 onwards, allowed the partial restoration of the original genetic nature of Lake Geneva's population and its levels of diversity (lesser genetic differentiation between current and original fishes, and similar levels of *H*_o_, *A*_r_, and F_is_). Accordingly, decades of supportive breeding involving local broodstock seem to have had little impact on the genetic composition in other fishes (Eldridge & Killebrew, 2008; Gow et al., 2011; Heggenes et al., 2006; Small, Currens, Johnson, Frye, & Von Bargen, 2009; Stelkens et al., 2012). In some cases, genetic diversity was even found to increase following such restocking practices, when lake populations are highly structured spatially and may admix with releases obtained from breeders caught in close‐by, yet genetically different source populations (Marie et al., 2010; Valiquette et al., 2014). Our results thus even suggest that conservation‐based supportive breeding may in fact serve as a one restoration measure to counteract the negative genetic effect of previous restocking involving exotic broodstocks. However, note that the conservation‐based breeding practice was still not sufficient to fully restore the original gene pool: contemporary individuals are still partly genetically differentiated from original ones (Figure 1b, Figure 2 right), as some native alleles were likely lost by drift and as others, gained through admixture with exotic fishes, presumably persist. Here, the higher contemporary allelic richness compared to the 1990s despite stocking by local fishes is somehow unexpected. In link with the stronger genetic structure in early stages of the program, it may reflect that all exotic alleles have since spread throughout the lake and were thus more exhaustively sampled recently.

On the other hand, the continuous restocking clearly led to the rapid and strong homogenization of the lake's population (toward null *F*_st_): even if the original genetic structure was low to begin with, it is virtually inexistent nowadays. This pattern is in line with previous studies on salmonids (Halbisen & Wilson, 2009; Lamaze et al., 2012; Marie et al., 2010). The ecological effects of such homogenization, and especially whether it hindered the local adaptation of Lake Geneva's population, are unclear. This aspect may be one of the working hypotheses for the long‐term decline of the Arctic charr in this lake, despite the intense restocking efforts. In any case, the issue of genetic homogenization must be considered to improve conservation‐based restocking strategies, notably by representing as many natural spawning sites among artificially reared broodstocks, and controlling their releases at their site of origins only.

Many studies have analyzed the genetic changes in restocked fish populations in space and time, exploiting historical samples (e.g., Gow et al., 2011; Gum, Geist, Eckenfels, & Brinker, 2014; Hansen, 2002; Heggenes et al., 2006; Nielsen et al., 1999; Perrier et al., 2013). However, to the best of our knowledge, our study is the first to provide the opportunity and the resolution to understand how the shift in restocking strategies influenced the genetic composition of populations. We show that the Arctic charr responded significantly, and very rapidly to these changes, possibly also due to the high intensity of stocking (which correlates to the amount of genetic changes; Valiquette et al., 2014), and the important contribution of stocked fishes in the population (45%–90% depending on age class; Caudron et al., 2014). Importantly, we also show that the diversity drop and genetic displacement induced by nonlocal stocking can be (at least partly) reversible with local supportive breeding. Given the improvement of restocking practices, combined with the growing need to artificially support declining wild stocks, similar genetic changes must have occurred in many exploited fish populations. Their consequences should be considered when evaluating current strategies, and implementing new ones.

### 4.3 Perspective for management of the Arctic charr in Lake Geneva

The intense restocking program of the Arctic charr in Lake Geneva was developed to reverse the continuous population decline observed in the 1970s, presumably due to eutrophication and pollution of the lake, as observed elsewhere across the Alpine region (Anneville, Gammeter, & Straile, 2005; Brunner et al., 1998; Caudron et al., 2014; Englbrecht, Schliewen, & Tautz, 2002; Lang, 1985, 1999). The program fulfilled its purpose up until the mid‐1990s, with the number of catches increasing proportionally to the restocking effort (Champigneulle & Gerdeaux, 1995). However, the situation drastically changed from 1999 onwards: Both professional and recreational catches severally dropped and the population appears no longer sustainable. Several factors were proposed to account for this unexpected decline, including predation, competition, climate fluctuations, micro‐pollutants, and genetic factors. A recent capture–mark–recapture study demonstrated that although stocked fishes were abundantly caught at immature stages (84% of two‐year‐old catches), twice less reached sexual maturity (44% of four‐year‐old catches), further questioning the efficiency of the program (Caudron et al., 2014). Indeed, these numbers suggest that fishes naturally born in the lake are rare but that stocked fishes often fail to reproduce. In parallel, substantial efforts of habitat recovery, especially at spawning sites, were successfully performed across the lake, so this should not be a contributing factor of current decline (Caudron et al., 2014; Rubin, 2005).

Thus, from our results, the genetic factors could be one plausible cause. First, although diversity seems back to its original level, the altered genetic composition and strong homogenization we observe across the lake might have diminished the fitness of fishes, through maladaptation and outbreeding depression following admixture (e.g., Le Cam et al., 2015). This effect may be exacerbated in stocked fishes, given the limited proportion that actually reaches sexual maturity (Caudron et al., 2014). Second, the stocks of one of the two hatcheries are facing inbreeding (*F*_is_ = 0.124 for FVCH), probably following rearing practices. This might translate into an intrinsically lower fitness of artificially bred individuals and compromise their fate in the wild.

In addition to restore and/or maintain suitable spawning conditions (fresh gravel and oxygen availability at deeper layers of the lake; Mari et al., 2016), we recommend two major lines of action to preserve the genetic make‐up of this population and limit undesirable genetic effects. First, restocking should be carried out locally, that is, by releasing fishes at their spawning sites of origin, in order to limit homogenization. In Lake Geneva, releases are conducted independently to the place of egg collection. Moreover, it should especially not involve strains from surrounding lakes, as commonly practiced elsewhere (Marie et al., 2010; Valiquette et al., 2014). Second, restocking protocols should increase the number of adults contributing to artificial broodstocks, in order to better represent the variation present in the natural population, as well as to limit inbreeding among released fishes.

## 5 CONCLUSIONS

Our study presents a comprehensive genetic survey reporting the long‐term effects of two different stocking practices of the Arctic charr in Lake Geneva, applied in turns. Our fine spatial and temporal resolutions allowed to dissect the detrimental genetic effects of historical restocking by nonlocal fishes, in accordance with previous work. Importantly, we could further show that supportive breeding of local fishes allowed to partially restore the genetic integrity of this altered population, but that current practices could be optimized to improve the restocking actions, and thus for this population to reach self‐sustainability.

## CONFLICT OF INTEREST

None declared.

## DATA ARCHIVING

Data for this study are available at the Dryad Digital Repository: http://dx.doi.org/10.5061/dryad.f776h.
