# Supplementary material for: Stocking activities for the Arctic charr in Lake Geneva: Genetic effects in space and time
Source: Ecol Evol. 2017 Jun 7;7(14):5201–11. doi: 10.1002/ece3.3073 (PMC5528235; doi:10.1002/ece3.3073)

Supporting information

Table S1: Information on microsatellites and PCR conditions. T_a: annealing temperature (°C); Group: group for post-PCR multiplexing genotyping; N_a: number of alleles

Locus	Ref.	T_a	PCR cycles	$MgCl_2$ (mM)	Group	Range	Na	Dye
CoCl-3	1	51	35	1.5	1	210-277	21	ATTO550
Ssa456	2	51	35	1.5	1	197-218	11	HEX
OtsG253b	3	55	35	1.5	1	80-122	10	FAM
Sfo-23	4	51	40	1.5	2	130-221	19	ATTO550
Ssa85	5	51	40	1.5	2	163-192	13	FAM
MST-85	6	51	35	2.5	3	211-272	29	ATTO550
Omy301	7	51	45	1.5	3	108-158	13	HEX
Sco19	8	51	40	1.5	3	183-191	5	FAM

[1] Bernatchez (1996) Rapport de l'université Laval à la vice-présidence Environnement et Collectives d'Hydro-Québec. [2] Slettan et al. (1997) Heredity 78 :620-627. [3] Williamson et al. (2002) Molecular Ecology Resources 100:17-19. [4] Angers et al. (1995) Journal of Fish Biology 47:177-185. [5] O'Reilly et al. (1996) Canadian Journal of Fisheries and Aquatic Sciences 53:2292-2298. [6] Presa & Guyomard (1996) Journal of Fish Biology 49:1326-1329. [7] Jackson et al. (1998) Heredity 80:143-151. [8] Taylor et al. (2001) Canadian Journal of Fisheries and Aquatic Sciences 58:406-420.

Table S2a: Population genetic indices: Allelic richness A_r (scaled to 9 individuals)

Locus:	CoCl3	Ssa456	OtsG253b	Sfo-23	Ssa85	MST-85	Omy301	Sco19	**average**
Contemporary samples									
CCH	7.0	4.4	5.2	6.6	5.1	11.2	4.7	1.5	**5.7**
VCH	6.9	4.2	5.7	6.2	4.7	10.2	5.6	2.4	**5.7**
MCH	6.3	4.5	4.1	7.1	5.5	10.5	6.0	1.7	**5.7**
BCH	5.9	5.5	5.0	7.6	6.1	11.4	6.2	1.5	**6.1**
YF	7.0	4.3	6.4	8.3	5.8	10.2	5.2	2.4	**6.2**
RF	8.0	4.2	4.6	6.9	6.4	12.4	7.3	1.5	**6.4**
LF	5.8	3.5	4.9	6.5	5.9	10.2	4.7	2.7	**5.5**
MFa	8.0	4.1	4.5	8.6	6.6	12.0	5.7	2.4	**6.5**
MFb	7.0	4.2	4.4	7.0	5.3	10.3	6.6	2.3	**5.9**
FVCH	8.4	5.6	4.4	8.7	6.9	11.5	6.2	1.5	**6.7**
FRF	6.4	4.5	4.6	8.3	5.1	10.8	5.5	1.8	**5.9**
Historical samples									
RF63	9.4	6.0	3.7	5.3	4.8	12.1	5.8	3.4	**6.3**
RF91	5.2	3.7	4.4	5.0	3.6	9.0	3.0	1.0	**4.4**
LF77	7.0	7.1	5.4	3.8	5.4	9.3	5.3	2.0	**5.7**
LF86	3.8	3.4	3.4	4.0	4.3	10.5	3.2	1.0	**4.2**
LF94	3.8	3.4	2.8	3.3	4.3	10.6	2.9	1.0	**4.0**
MF60	6.6	5.6	5.7	4.8	5.4	10.4	3.5	2.0	**5.5**
MF75	6.8	4.4	5.2	5.3	5.7	9.3	6.3	2.0	**5.6**
MF86	4.9	5.2	4.2	2.7	4.6	10.0	4.5	2.0	**4.8**
MF94	8.2	3.9	4.4	5.2	5.0	8.8	6.3	1.5	**5.4**
**average**	**6.6**	**4.6**	**4.7**	**6.0**	**5.3**	**10.5**	**5.2**	**1.9**	

Table S2b: Population genetic indices: Observed heterozygosity H_o.

Locus:	CoCl3	OtsG253b	Ssa456	Sfo-23	Ssa85	MST-85	Omy301	Sco19	**average**
Contemporary samples									
CCH	0.74	0.70	0.73	0.76	0.64	0.94	0.62	0.05	**0.65**
VCH	0.84	0.63	0.71	0.75	0.69	0.91	0.74	0.19	**0.68**
MCH	0.75	0.43	0.68	0.84	0.62	0.93	0.77	0.10	**0.64**
BCH	0.74	0.65	0.80	0.84	0.72	0.94	0.78	0.05	**0.69**
YF	0.83	0.79	0.72	0.84	0.70	0.92	0.73	0.23	**0.72**
RF	0.85	0.55	0.71	0.78	0.72	0.95	0.81	0.05	**0.68**
LF	0.76	0.54	0.70	0.82	0.64	0.93	0.69	0.32	**0.68**
MFa	0.82	0.53	0.68	0.79	0.70	0.95	0.79	0.19	**0.68**
MFb	0.78	0.50	0.70	0.77	0.66	0.92	0.77	0.19	**0.66**
FVCH	0.82	0.57	0.77	0.89	0.76	0.94	0.78	0.06	**0.70**
FRF	0.75	0.64	0.75	0.87	0.59	0.93	0.70	0.14	**0.67**
Historical samples									
RF63	0.89	0.48	0.74	0.54	0.71	0.95	0.69	0.31	**0.66**
RF91	0.68	0.66	0.69	0.44	0.63	0.90	0.56	0.00	**0.57**
LF77	0.83	0.71	0.81	0.68	0.67	0.88	0.68	0.39	**0.71**
LF86	0.49	0.56	0.65	0.49	0.55	0.94	0.57	0.00	**0.53**
LF94	0.58	0.51	0.60	0.45	0.63	0.94	0.52	0.00	**0.53**
MF60	0.71	0.70	0.79	0.62	0.64	0.91	0.63	0.37	**0.67**
MF75	0.81	0.68	0.73	0.75	0.70	0.90	0.75	0.36	**0.71**
MF86	0.61	0.64	0.73	0.45	0.68	0.93	0.60	0.22	**0.61**
MF94	0.80	0.64	0.68	0.70	0.61	0.87	0.79	0.05	**0.64**
**average**	**0.75**	**0.60**	**0.72**	**0.70**	**0.66**	**0.92**	**0.70**	**0.16**	

Table S2c: Population genetic indices: Inbreeding coefficient F_{is}. *: significantly departing from 0 for the corresponding population (after Bonferroni correction for multiple tests).

Locus:	CoCl3	OtsG253b	Ssa456	Sfo-23	Ssa85	MST-85	Omy301	Sco19	**All**
Contemporary samples									
CCH	0.00	-0.08	-0.23	0.10	0.30	0.04	-0.05	0.00	**0.01**
VCH	-0.13	-0.04	0.01	0.26	-0.09	0.06	-0.09	-0.06	**0.00**
MCH	0.02	-0.05	0.15	0.17	0.03	0.06	-0.16	-0.03	**0.03**
BCH	0.12	0.02	-0.04	0.16	0.09	-0.06	0.11	0.00	**0.05**
YF	-0.14	-0.02	0.38	-0.06	0.00	-0.09	0.18	-0.09	**0.02**
RF	0.00	-0.18	-0.13	-0.03	-0.05	0.00	-0.11	0.00	**-0.06**
LF	-0.03	0.18	0.12	0.03	-0.15	-0.08	0.44	-0.15	**0.05**
MFa	0.02	-0.04	0.26	0.14	0.00	0.00	-0.14	-0.06	**0.03**
MFb	0.17	-0.21	0.10	0.03	0.09	0.26	-0.23	-0.06	**0.04**
FVCH	0.11	0.08	0.25	0.23	-0.11	0.05	0.25	0.00	**0.12***
FRF	-0.06	-0.16	0.07	0.02	-0.10	0.14	-0.21	-0.06	**-0.03**
Historical samples									
RF63	0.21	-0.21	-0.01	0.33	-0.15	0.05	0.28	-0.09	**0.07**
RF91	-0.39	-0.14	-0.09	-0.03	-0.51	-0.12	-0.60	NA	**-0.26***
LF77	-0.20	-0.42	-0.13	-0.35	-0.24	0.06	0.20	-0.29	**-0.16***
LF86	-0.23	-0.26	-0.31	-0.23	-0.28	0.20	-0.76	NA	**-0.23***
LF94	-0.37	-0.29	-0.01	-0.23	-0.26	0.16	-0.53	NA	**-0.18***
MF60	0.25	-0.09	-0.18	0.06	0.08	0.09	-0.21	-0.28	**-0.02**
MF75	-0.04	-0.18	0.05	0.13	0.08	0.28	-0.26	-0.27	**0.00**
MF86	-0.31	0.06	-0.10	0.18	-0.19	0.03	-0.25	-0.12	**-0.09**
MF94	-0.05	-0.15	0.15	0.47	0.06	0.33	0.20	0.00	**0.15***
**average**	**-0.05**	**-0.11**	**0.02**	**0.07**	**-0.07**	**0.07**	**-0.10**	**-0.09**	

Table S2d: Population genetic indices: Pairwise genetic differentiation F_{st}. In bold: significantly departing from 0 (after Bonferroni correction for multiple tests). Blue: contemporary samples; Green: samples prior to stocking (before 1979); Red: samples in early stages of stocking (1986-1994).

	VCH	MCH	BCH	YF	RF	LF	MFa	MFb	FVCH	FRF	RF63	LF77	MF60	MF75	RF91	LF86	LF94	MF86	MF94
CCH	0.01	0.01	0.00	0.00	0.00	0.01	0.01	0.01	0.01	0.00	0.01	**0.04**	0.02	0.02	0.02	**0.03**	**0.04**	**0.04**	0.00
VCH	-	0.01	0.00	0.01	0.00	0.00	0.00	0.00	0.00	0.01	0.02	**0.04**	0.02	0.01	**0.03**	**0.05**	**0.05**	**0.03**	0.00
MCH		-	0.01	0.02	0.00	0.00	0.00	0.00	0.00	0.00	0.02	0.06	**0.04**	**0.03**	**0.05**	**0.06**	**0.07**	**0.04**	0.03
BCH			-	0.00	0.00	0.00	0.00	0.01	0.00	0.00	0.02	0.03	0.01	0.01	**0.02**	**0.03**	**0.03**	**0.03**	0.00
YF				-	0.00	0.00	0.01	0.01	0.00	0.00	0.02	0.02	0.01	0.01	**0.05**	**0.06**	**0.06**	**0.04**	0.01
RF					-	0.00	0.00	0.00	0.00	0.00	0.00	0.03	0.02	0.01	0.03	0.04	0.04	0.03	0.00
LF						-	0.00	0.00	0.00	0.00	0.01	0.03	0.01	0.01	**0.04**	**0.04**	**0.04**	0.02	0.01
MFa							-	0.01	0.00	0.00	0.00	0.04	0.02	0.01	0.03	**0.04**	**0.03**	0.02	0.00
MFb								-	0.00	0.00	0.01	**0.04**	0.02	0.02	**0.04**	**0.04**	**0.05**	**0.03**	0.02
FVCH									-	0.00	0.01	0.03	0.02	0.01	**0.04**	**0.04**	**0.04**	0.03	0.00
FRF										-	0.02	**0.04**	0.02	**0.02**	**0.04**	**0.05**	**0.05**	**0.04**	0.01
RF63											-	0.03	0.02	**0.01**	0.02	0.04	0.03	0.02	0.02
LF77												-	0.01	0.01	**0.08**	**0.08**	**0.08**	**0.06**	0.03
MF60													-	0.00	**0.02**	**0.03**	**0.02**	0.01	0.00
MF75														-	**0.03**	**0.04**	**0.04**	**0.03**	0.01
RF91															-	**0.03**	**0.02**	**0.03**	**0.02**
LF86																-	0.01	**0.03**	**0.02**
LF94																	-	**0.02**	0.02
MF86																		-	0.02

Fig. S1: Tree of genetic distance (F_{st}) among Lake Geneva spawning sites. Blue: contemporary samples; green: prior to restocking; red: in early stages of restocking

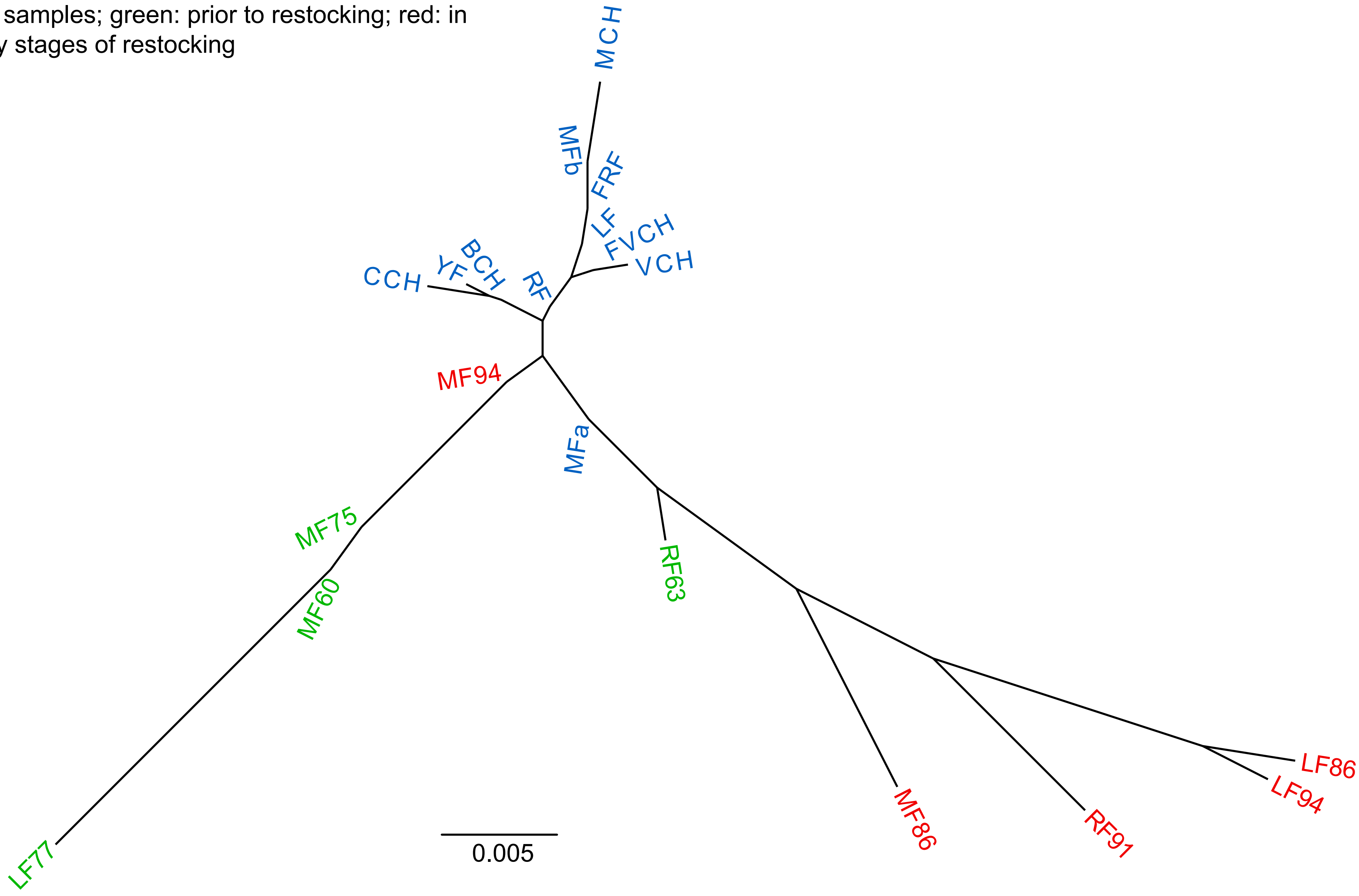

Supplement: Supplementary file 1 [file ECE3-7-5201-s001.pdf]
